# The impact of core training combined with breathing exercises on individuals with chronic non-specific low back pain

**DOI:** 10.3389/fpubh.2025.1518612

**Published:** 2025-02-21

**Authors:** Ying Li, Qian Zhao, Xiao Zhang, Yan E, Yuqin Su

**Affiliations:** ^1^School of Physical Education, Qingdao University of Science and Technology, Qingdao, China; ^2^ChongQing Finance and Economics College, Chongqing, China; ^3^Qingdao Municipal Sports Bureau, Qingdao, China; ^4^Institute of Sport Science, College of Physical Education, Southwest University, Chongqing, China

**Keywords:** chronic non-specific low back pain, core training, breathing exercises, exercise therapy, pain management, functional improvement

## Abstract

**Background:**

Chronic non-specific low back pain (CNLBP) is a common condition, defined as pain lasting more than 3 months between the lower thoracic margin and gluteal folds, without identifiable tissue damage. Despite its low disability rate, the complex etiology and high recurrence impose significant health and socioeconomic burdens. According to European LBP guidelines, exercise therapy is the preferred treatment for CNLBP. This study evaluates the efficacy of core training combined with breathing exercises as a therapeutic intervention for CNLBP.

**Methods:**

Eighteen CNLBP patients were randomly assigned to three groups: core training only, core training with breathing exercises, and a control group. A 12-week intervention included VAS, ODI scores, and muscle strength tests.

**Results:**

The combined group showed significantly greater pain reduction, functional improvement, and muscle strength enhancement compared to the other groups.

**Conclusion:**

Core training with breathing exercises is more effective in alleviating CNLBP symptoms, highlighting the added value of integrating breathing exercises.

## 1 Introduction

Chronic non-specific low back pain (CNLBP) is one of the leading global health issues, significantly affecting individual quality of life and socioeconomic systems (1–3). The pathogenesis of CNLBP is a pathological process where internal and external factors disrupt the balance of biomechanical subsystems, leading to dysfunction in muscles, ligaments, joints, and nerves (4–6). Epidemiological studies indicate that approximately 84% of individuals worldwide experience low back pain (LBP) during their lifetime, with 85% of cases classified as non-specific (6). Among these, 5–10% of non-specific cases persist beyond 12 weeks and develop into chronic pain, while 90–95% of acute cases resolve within the same period (7). Globally, the annual incidence of LBP is 245.9 million cases, with a prevalence of 577 million cases, accounting for 3.2 and 7.6% of the total population, respectively, and this burden has increased by 50% over the past 20 years. LBP is most prevalent among individuals aged 40–50 years and is more common in high Socio-Demographic Index (SDI) countries, where the risk is over three times higher than in low-SDI countries. Projections suggest that by 2050, the incidence, prevalence, and disability-adjusted life years (DALYs) associated with LBP will increase by approximately 1.4 times (8). In the United States, the annual direct medical expenditure for CNLBP amounts to $33 billion, with indirect costs reaching $90 billion and a disability rate of 11–12% (9, 10). These data underscore the critical importance of early intervention to mitigate the risk of acute cases progressing to chronic conditions.

However, effective treatment strategies must also consider psychological factors such as pain catastrophizing, kinesiophobia, and emotional distress, which have been shown to influence pain perception and the chronicity of the condition (11). These psychosocial factors can amplify the impact of physical symptoms, hinder recovery, and contribute to the persistence of CNLBP. Therefore, incorporating interventions that target stress reduction and emotional regulation is crucial for improving patient outcomes and enhancing the effectiveness of biomechanical treatments.

Existing treatments, including medications, physical therapy, and surgical interventions, are effective in alleviating symptoms but often fail to provide lasting benefits due to their short-term effects and potential side effects. For example, medications such as NSAIDs and opioids can provide temporary pain relief but are associated with gastrointestinal problems, cardiovascular risks, dependency, and sedation (9, 12). Physical therapy, including manual therapy and exercises, often helps relieve symptoms temporarily but can cause mild discomfort, minor injuries, and typically fails to address biomechanical issues such as poor posture, muscle imbalances, or spinal instability (8, 12). Surgical interventions, while appropriate in specific cases, carry risks such as infection, nerve damage, and persistent pain, and do not adequately target the functional or biomechanical causes of non-specific low back pain (9, 12). These limitations highlight the urgent need for more integrative and sustainable therapeutic strategies that address both symptomatic relief and underlying biomechanical contributors to chronic pain.

Exercise therapy is recognized for its potential to offer long-term symptomatic improvement by focusing on strengthening core muscles, enhancing lumbar stability, and promoting a healthier lifestyle (13). Specific exercise methodologies, such as William’s Flexion Exercise, focus on stretching and strengthening the lumbar muscles to reduce spinal load and alleviate pain (14). However, they are often unsuitable for patients with lumbar disk protrusions (15). The McKenzie Extension Exercise regimen emphasizes self-management and preventive tactics, tailoring interventions to individual pain profiles (16). Nevertheless, it does not fully address core stability or respiratory coordination. Pilates aims to enhance overall flexibility, core strength, and postural alignment through apparatus-assisted and mat-based exercises (Huang et al., 2024), which help improve lumbar-pelvic stability. However, these tends to overlook the importance of breathing coordination (17). Despite their effectiveness, these approaches do not comprehensively address the complex, multifactorial nature of CNLBP.

More recent studies have underscored the potential effectiveness of core training and breathing exercises in overcoming the limitations of individual therapies for CNLBP. Core training plays a critical role in strengthening muscles essential for lumbar and trunk stability, contributing to pain alleviation and improved spinal function (11, 18–20). Breathing exercises, on the other hand, primarily focus on enhancing diaphragm function and increasing intra-abdominal pressure, mechanisms known to alleviate pain and support lumbar stability (21, 22). While these two modalities have individually demonstrated significant benefits, the integration of both approaches remains underexplored in the context of CNLBP. Although research has confirmed the effectiveness of these individual exercises (17, 18), no comprehensive study has yet combined these approaches to address the multifaceted nature of CNLBP, particularly in terms of both biomechanical and psychological factors. This gap in the literature highlights the need for our study. These gaps in the literature provide a rationale for the present study, which integrates core training with breathing exercises to offer a more holistic and comprehensive intervention strategy for CNLBP. By targeting both mechanical and psychological components, this research proposes an innovative approach aimed at addressing the physical limitations of the condition while also considering its emotional and cognitive aspects. The study evaluates the combined effects of core training and breathing exercises through various outcome measures, including pain intensity, lumbar function, and core muscle strength. This integrated approach is expected to provide new insights into improving both functional and psychological outcomes in CNLBP, thus paving the way for more effective therapeutic strategies.

## 2 Materials and methods

### 2.1 Participants

This randomized controlled trial (RCT) aimed to evaluate the effects of core training and combined training on chronic low back pain. The study recruited 18 participants with unilateral low back pain based on specific inclusion and exclusion criteria. Approved by the Ethics Review Committee at Southwest University (SWU-PE-20230928), the inclusion criteria were as follows: (1) age 18–60 years; (2) low back pain duration ≥3 months (23), which is the standard threshold used to define chronic low back pain according to clinical guidelines and previous studies (23); (3) no organic lumbar diseases; (4) unilateral lumbar pain; (5) ability to undergo a 12-week exercise intervention; (6) no recent therapy or interventions. Exclusion criteria included: (1) abnormal imaging findings; (2) spinal surgery history; (3) severe chronic diseases; (4) visceral disease-related pain; (5) VAS > 8 points; (6) severe lumbar diseases, pregnancy, or breastfeeding.

Given the small sample size (*n* = 18) in this study, we assessed the normality of the data using the Shapiro–Wilk test. The results indicated that some variables, including age, weight, and duration of illness, did not follow a normal distribution (*p*-values <0.05). As a result, we opted for non-parametric statistical tests. Additionally, we evaluated the homogeneity of variances using Levene’s test, and the results showed no significant differences in variances between groups (*p*-values >0.05). To compare the groups across various variables, we used the Kruskal-Wallis test. The results indicated that there were no significant differences between the groups for age, height, weight, BMI, and duration of illness (*p*-values >0.05). Therefore, we can confirm that the data meet the assumptions of homogeneity of variances, and the statistical analyses are valid (Table 1).

**Table 1:** Shapiro–Wilk test, Levene’s test and Kruskal-Wallis test for Participants [*N* = 18].

Variables	Shapiro–Wilk statistic (*N* = 18)	*p*	Levene’s test (*N* = 18)	*p*	Kruskal-Wallis test (*N* = 18)	*p*
Age	0.78	0.00	2.57	0.11	0.19	0.91
Height	0.94	0.30	1.39	0.28	0.60	0.74
Weight	0.88	0.03	0.56	0.58	1.29	0.53
BMI	0.98	0.96	0.66	0.53	0.02	0.99
Duration of illness	0.87	0.02	0.01	0.99	3.26	0.20

Significance *p* < 0.05.

The participants were randomly assigned to one of three groups—core training only (Group A, 6 participants), combined training (Group B, 6 participants), and the control group (Group C, 6 participants, no intervention)—using a computer-generated random sequence. This approach ensured equitable allocation of participants. To further minimize selection bias, block randomization was applied, which not only maintained equal group sizes but also ensured balance across the groups throughout the study.

Although baseline characteristics showed no significant differences between groups (Table 2), the participants were recruited from a similar region, occupation, and age range, which may have contributed to the lack of significant variation. This homogeneity, alongside the small sample size, could explain the absence of baseline differences, while the rigor of the randomization process ensures reliable results.

**Table 2:** Basic data statistics of the participants [*N* = 18].

	Group A	Group B	Group C	*p*
Variables	(*n* = 6, 2 males, 4 females)	(*n* = 6, 2 males, 4 females)	(*n* = 6, 1 males, 5 females)	
Age (year)	25.14 ± 1.35	23.83 ± 2.48	30.33 ± 7.61	0.06
Height (m)	1.67 ± 0.85	1.65 ± 0.51	1.67 ± 0.13	0.93
Weight (kg)	58.00 ± 10.07	54.5 ± 6.19	59.17 ± 8.79	0.66
BMI(kg/m^2^)	20.70 ± 1.98	19.95 ± 1.69	21.11 ± 1.17	0.49
Duration of illness (months)	7.86 ± 2.33	7.83 ± 3.71	8.83 ± 3.76	0.88

Group A: Core Training, Group B: Core + Breathing, Group C: Control, significance *p* < 0.05.

The study was conducted as a double-blind experiment. Participants were informed of the study’s procedures, risks, and benefits, and all provided written informed consent.

### 2.2 Measurement tools

#### 2.2.1 Visual analog scales

VAS, a 10 cm pain assessment tool, is reliable and valid for CNLBP patients (24). A 30% improvement from baseline represents the MCID, ensuring clinical relevance (25).

#### 2.2.2 Oswestry disability index

The ODI assesses low back pain-related disability and is validated for CNLBP (26). Its MCID of 30% aids in evaluating significant treatment effects (27).

#### 2.2.3 Core muscle strength testing

MicroFET3™, a reliable tool for core strength measurement, has been validated for CNLBP (18, 28). It provides precise peak force data for evaluating training outcomes (line x, page x).

### 2.3 Study design

This 12-week (29, 30) randomized controlled trial assesses the effects of exercise interventions on individuals with CNLBP. Participants are randomly assigned to three groups: Core Training (Group A), Core Training with Breathing Exercises (Group B), and Control (Group C). Group A undergoes core stability training, while Group B combines core training with breathing exercises to enhance lumbar muscle coordination. Group C maintains their usual lifestyle without intervention, providing a baseline for comparison.

The randomization process was carried out using a computer-generated random sequence, and block randomization was used to ensure equal group sizes. The 12-week duration was selected based on previous research, allowing sufficient time for observable effects while minimizing dropout. To ensure adherence, participants were regularly monitored through online community supervision and weekly check-ins.

**Figure 1:**
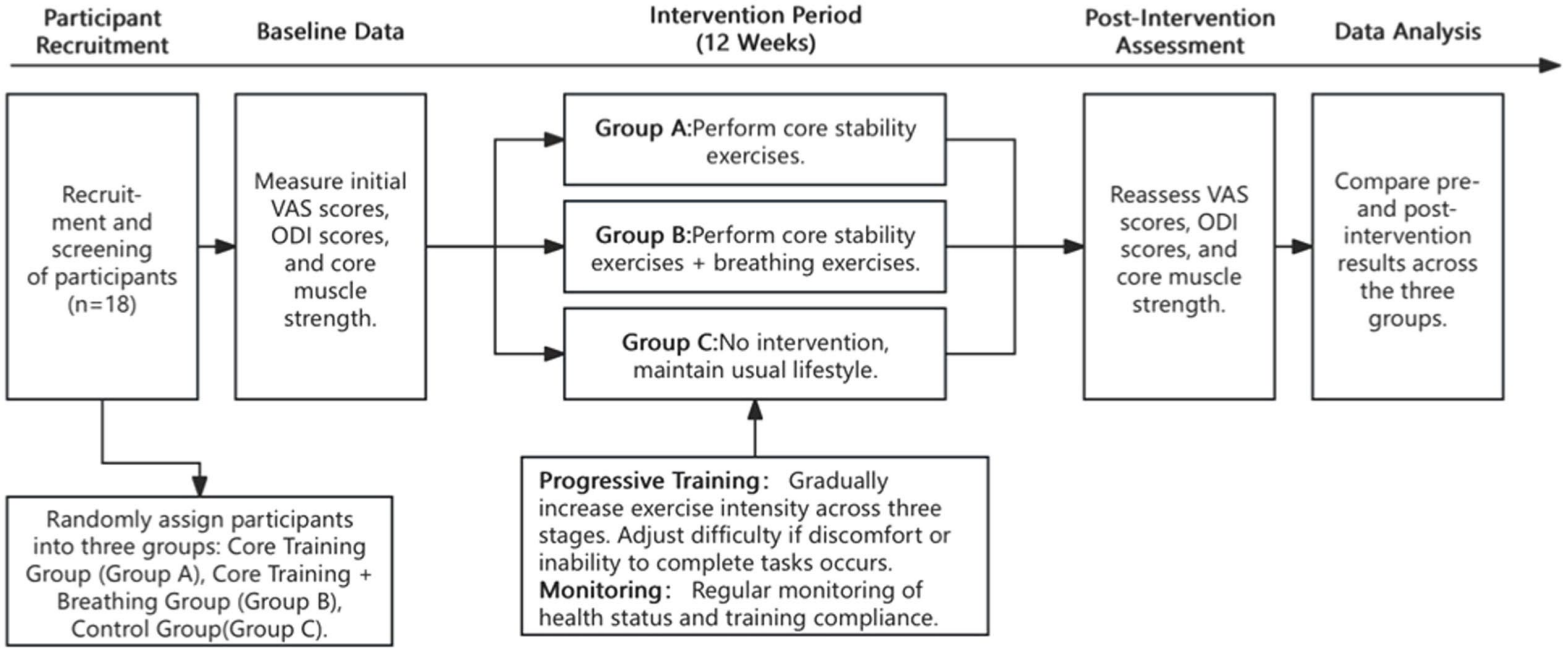
Flow chart of the experimental procedure.

Training intensity is progressively increased, and adjustments are made if participants experience discomfort. The study monitors participants’ health and training activities, comparing pre- and post-experiment VAS, ODI scores, and core muscle strength to evaluate the effectiveness of the interventions (Figure 1).

### 2.4 Exercise intervention

Each group underwent 40-min sessions (5-min warm-up +35-min exercise), 3 times per week for 12 weeks, with intensity adjusted based on feedback.

#### 2.4.1 Core training only group (group A)

##### 2.4.1.1 Phase 1 (weeks 1–4): bodyweight core stability exercises

Objective: Activate core muscles, improve proprioception, and learn correct movement patterns.

Frequency: 3 sessions per week, 40 min each.

Warm-up: 5 min of light jogging or bodyweight exercises.

Exercises:

(1) Crunches: 1 min × 3 sets (30s rest).
(2) Bicycle Exercise: 1 min × 3 sets (30s rest).
(3) Glute Bridge: 1 min × 3 sets (30s rest).
(4) Mason Twist: 1 min × 3 sets (30s rest).
(5) Abdominal Stretch: 5 min.

##### 2.4.1.2 Phase 2 (weeks 5–8): Swiss ball-assisted exercises

Objective: Engage deep trunk muscles and improve core stability under unstable conditions.

Frequency: 3 sessions per week, 40 min each.

Warm-up: 5 min of light jogging or bodyweight exercises.

Exercises:

(1) Supine Position: 1 min × 3 sets (1 min rest).
(1) Seated on Swiss Ball: 1 min × 3 sets (1 min rest).
(1) Prone Position with Swiss Ball: 1 min × 3 sets (1 min rest).
(1) Reverse Bridge: 30 s × 10 reps.
(1) Hip Flexion: 15 s per side × 10 reps.
(1) Single-Leg Balance: 15 s per side × 10 reps.
(1) Stretching: 5 min.

##### 2.4.1.3 Phase 3 (weeks 9–12): advanced core stability with weighted equipment

Objective: Enhance core strength and stability with added resistance, improving neuromuscular control.

Frequency: 3 sessions per week, 40 min each.

Warm-up: 5 min of light jogging or bodyweight exercises.

Exercises:

(1) Weighted Back Lift: 1 min × 3 sets (1 min rest).
(2) Side Raises with Dumbbells: 1 min × 3 sets (1 min rest).
(3) Weighted Crunches: 1 min × 3 sets (1 min rest).
(4) Weighted Mason Twists: 1 min × 3 sets (1 min rest).
(5) Weighted Leg Raises: 1 min × 3 sets (1 min rest).
(6) Stretching: 5 min.

#### 2.4.2 Core training combined with breathing exercises group (group B)

Objective: To combine core stability training with breathing exercises to enhance lumbar muscle coordination and respiratory control.

Duration:

Total training time: 40 min.

Core training: 30 min.

Breathing exercises: 10 min.

Frequency: 3 sessions per week.

##### 2.4.2.1 Phase 1: core training (30 min)

The core training exercises for this group follow the same routine as those used in the Core Training Only group, with the same set duration, repetitions, and rest periods.

Breathing exercises (10 min).

Breathing exercises are incorporated into the core training sessions at the end of each core training set. The following three exercises are performed.

##### 2.4.2.2 Exercise 1: abdominal breathing in supine, kneeling, and standing positions

Objective: To activate diaphragmatic breathing and enhance lung capacity.

Method: Begin by placing both hands gently under the ribs, fingers pointing forward. When diaphragmatic breathing is used effectively, the ribs should expand laterally and anteriorly, while the abdomen should protrude. If the chest rises, the diaphragm is not being used correctly—relax the shoulders and chest.

Frequency: 20 breaths per position, 3 sets.

Positions: Supine, kneeling, and standing.

##### 2.4.2.3 Exercise 2: balloon breathing

Objective: To strengthen diaphragmatic control and improve respiratory muscle coordination.

Method: The participant sits with hands resting on their knees, without using hands to assist with holding the balloon. The balloon is placed in the mouth, and the participant inhales through the nose and exhales into the balloon until exhalation is complete. The participant holds their breath for 5 s after exhalation.

Frequency: 3 sets of 5 repetitions.

##### 2.4.2.4 Exercise 3: supine balloon breathing

Objective: To further activate the diaphragm and engage the pelvic floor muscles in coordination with the core.

Method: The participant lies supine with feet pressed against the wall, knees bent at 90 degrees, and a foam roller placed between the inner thighs. The participant raises one leg from the wall and extends the opposite arm while maintaining the breathing technique from Exercise 1.

Frequency: 3 sets of 3 repetitions per side.

#### 2.4.3 Control group (group C)

Maintained usual daily routines without exercise intervention for baseline comparison.

### 2.5 Statistical analysis

Statistical analysis was conducted using SPSS 25.0 and Excel 2016. Data were presented as mean ± SD. One-way ANOVA and LSD tests were used to analyze between-group differences, and paired *t*-tests were applied for within-group comparisons. Given the small sample size (*N* = 18), the Shapiro–Wilk test was employed to assess normality for all variables. For variables that did not meet the normality assumption, non-parametric methods (Mann–Whitney *U* test) were applied to ensure robust analysis. Randomization and block allocation were performed to minimize selection bias and ensure balanced group distributions. The significance level was set at *p* < 0.05.

## 3 Results

### 3.1 Comparison of the changes of Core muscle strength test

After the intervention, the Core Training Group showed significant improvements in muscle strength for lateral flexion (painful side) (*p* = 0.04) and rotation (non-painful side) (*p* = 0.01). The Core Training Combined with Breathing Exercises Group demonstrated significant improvements in flexion (*p* = 0.01), extension (*p* = 0.02), lateral flexion (non-painful side) (*p* < 0.01), and rotation (painful and non-painful sides) (*p* < 0.01). The Control Group showed no significant changes in any muscle strength indicators (Table 3). Pre-intervention, there were no significant between-group differences in muscle strength.

**Table 3:** Comparison of the changes of core muscle strength test.

		Before intervention	After intervention	*T*
Flexion (*N*)	a	134.09 ± 48.87	144.92 ± 47.78	*T* = −1.50, *p* = 0.19
	b	130.70 ± 24.02	154.10 ± 27.69	***T* = −4.14, *p* = 0.01**
	c	168.80 ± 62.00	157.80 ± 37.53	*T* = 0.89, *p* = 0.42
	F	*F* = 1.19, *p* = 0.33	*F* = 0.19, *p* = 0.83	
	LSD	a-b, b-c, a-c	a-b, b-c, a-c	
Extension (*N*)	a	118.23 ± 38.84	126.13 ± 39.62	*T* = −1.55, *p* = 0.17
	b	139.63 ± 31.08	153.10 ± 31.90	***T* = −3.28, *p* = 0.02**
	c	126.52 ± 29.72	123.67 ± 21.96	*T* = 0.83, *p* = 0.45
	F	*F* = 0.65, *p* = 0.53	*F* = 1.54, *p* = 0.24	
	LSD	a-b, b-c, a-c	a-b, b-c, a-c	
Lateral flexion (Painful) (*N*)	a	110.90 ± 33.08#	119.06 ± 36.42#	***T* = −2.64, *p* = 0.04**
	b	102.65 ± 14.00#	109.38 ± 14.87#	*T* = −1.82, *p* = 0.13
	c	107.10 ± 20.09#	105.67 ± 17.41#	*T* = 0.31, *p* = 0.77
	F	*F* = 0.58, *p* = 0.56	*F* = 0.48, *p* = 0.63	
	LSD	a-b, b-c, a-c	a-b, b-c, a-c	
Lateral flexion (Non-painful) (*N*)	a	123.03 ± 28.06#	132.85 ± 32.19#	*T* = −2.23, *p* = 0.07
	b	120.07 ± 11.92#	137.57 ± 11.25#	***T* = −5.96, *p* = 0.00**
	c	126.83 ± 24.83#	124.17 ± 25.10#	*T* = 0.92, *p* = 0.40
	F	*F* = 0.06, *p* = 0.93	*F* = 0.45, *p* = 0.65	
	LSD	a-b, b-c, a-c	a-b, b-c, a-c	
Rotation (Painful) (*N*)	a	88.23 ± 41.00#	97.60 ± 30.33	*T* = −2.00, *p* = 0.09
	b	86.35 ± 22.83	106.02 ± 20.96	***T* = −10.98, *p* = 0.00**
	c	118.48 ± 37.64	106.33 ± 22.99#	*T* = 1.31, *p* = 0.25
	F	*F* = 1.49, *p* = 0.24	*F* = 0.25, *p* = 0.78	
	LSD	a-b, b-c, a-c	a-b, b-c, a-c	
Rotation (Non-painful) (*N*)	a	96.61 ± 40.14#	107.07 ± 44.72	***T* =** −**3.63, *p* = 0.01**
	b	105.58 ± 13.62	128.77 ± 16.18	***T* =** −**7.11, *p* = 0.00**
	c	116.10 ± 19.88	125.5 ± 29.79#	*T* = −1.17, *p* = 0.30
	F	*F* = 1.44, *p* = 0.25	*F* = 0.82, *p* = 0.46	
	LSD	a-b, b-c, a-c	a-b, b-c, a-c	

‘a’ represents the Core Training Only Group, ‘b’ represents the Combined Group, and ‘c’ represents the Control Group. Asterisks (‘*’) indicate a significant difference (*p* < 0.05), while double asterisks (‘**’) indicate a highly significant difference (*p* < 0.01). Bold text indicates that the result is statistically significant.

### 3.2 Comparison of the changes of VAS

Before the intervention, VAS differences among groups were not significant. Post-intervention, VAS significantly decreased in the Combined Group (*p* < 0.01) and marginally in the Core Training Group (*p* = 0.06). No significant change occurred in the Control Group. Combined and Core Training groups had significantly lower VAS than the Control Group (*p* < 0.01, *p* < 0.05) (Table 4).

**Table 4:** Comparison of the changes of VAS.

	Before intervention	After intervention	*T*
a	4.14 ± 1.77	3.14 ± 1.35	*T* = 2.29, *p* = 0.06
b	5.17 ± 0.98	2.33 ± 0.82	***T* = 9.22, *p* = 0.00**
c	4.83 ± 0.75	5.16 ± 0.75	*T* = −0.67, *p* = 0.53
F	*F* = 1.08, *p* = 0.36	*F* = 12.08, *p* = 0.00	
LSD	a-b, b-c, a-c	a-b, b-c*, a-c*	

“a” represents the Core Training Only Group, “b” represents the Combined Group, and “c” represents the Control Group. “*F*” indicates the *F*-statistic used in the ANOVA test, “*T*” represents the *T*-statistic for comparing means, and “LSD” refers to the Least Significant Difference method used for post-hoc testing. The significance levels are indicated, where “*” represents a significant difference with *p* < 0.05 and “**” indicates a highly significant difference with *p* < 0.01. Bold text indicates that the result is statistically significant.

### 3.3 Comparison of the changes of ODI

Before the intervention, no significant differences were observed among the groups (Table 5). Post-intervention, the Core Training Only Group showed significant reductions in sitting (*p* < 0.05) and highly significant reductions in self-care, lifting, standing, activities, and overall scores (*p* < 0.01). The Combined Group had significant reductions in occupational/household activities (*p* < 0.05) and highly significant reductions in pain, self-care, lifting, sitting, standing, social activities, and overall scores (*p* < 0.01). The Control Group showed no significant changes. Both intervention groups had significantly lower post-intervention scores than the Control Group, with the Combined Group showing additional improvements (Table 5).

**Table 5:** Comparison of the changes of ODI.

	Before intervention			After intervention		
	a	b	c	a	b	c
Pain intensity	3.00 ± 1.15	3.50 ± 0.84	3.33 ± 0.52	2.14 ± 1.07	1.50 ± 0.55▲**	2.83 ± 0.75
Self-care ability	1.71 ± 0.95	2.33 ± 0.82	2.50 ± 0.55	1.00 ± 0.82▲**	0.67 ± 0.52▲**	1.83 ± 0.41
Lifting	2.71 ± 0.95	2.33 ± 0.52	1.83 ± 1.17	1.57 ± 0.79**	1.00 ± 0.89**	1.67 ± 0.82
Walking	0.00 ± 0.00	0.00 ± 0.00	0.33 ± 0.82	0.00 ± 0.00	0.00 ± 0.00	0.50 ± 1.22
Sitting	2.29 ± 1.38	3.00 ± 1.10	3.17 ± 0.75	1.43 ± 0.79▲*	1.17 ± 0.75▲**	2.50 ± 0.84
Standing	3.00 ± 0.82	2.33 ± 0.52	2.83 ± 0.75	1.86 ± 0.38★**	0.83 ± 0.41▲◆**	2.50 ± 0.84
Sleeping	1.14 ± 0.90	0.67 ± 0.52	1.50 ± 0.55	0.71 ± 0.49▲	0.17 ± 0.41▲	1.67 ± 1.21
Occupational/Household activities	2.00 ± 1.15	2.33 ± 0.82	2.83 ± 0.75	1.29 ± 0.95▲**	1.00 ± 0.89▲*	3.00 ± 0.63
Social activities	1.71 ± 1.11	2.17 ± 0.75	2.00 ± 0.89	0.57 ± 0.98▲**	0.50 ± 0.84▲**	2.33 ± 1.63
Travel	0.57 ± 0.53	0.83 ± 0.41	0.67 ± 0.52	0.57 ± 0.53	0.33 ± 0.52▲	1.00 ± 0.00
Total score	0.36 ± 0.12	0.39 ± 0.09	0.43 ± 0.09	0.22 ± 0.06★▲**	0.14 ± 0.06▲◆**	0.40 ± 0.06

“a” represents the Core Training Only Group, “b” represents the Combined Group, and “c” represents the Control Group. “*F*” refers to the *F*-statistic used in the ANOVA test, “*T*” refers to the *T*-statistic used to compare means, and “LSD” refers to the Least Significant Difference method used for post-hoc testing. The significance levels are indicated as follows: “*” indicates a significant difference (*p* < 0.05), “**” indicates a highly significant difference (*p* < 0.01), ▲ indicates a significant difference compared to the Control Group, ★ indicates a significant difference compared to the Combined Group, and ◆ indicates a significant difference compared to the Core Training Only Group.

## 4 Discussion

This study demonstrated that both core training and combined training significantly improved outcomes in patients with CNLBP. Notably, the combined training group achieved superior results in key areas, including pain reduction, functional enhancement, and core muscle strength improvements. Participants in this group experienced greater reductions in pain intensity and exhibited marked improvements in activities requiring postural stability, such as standing and traveling. These findings underscore the potential advantages of integrating breathing exercises into traditional core training, offering a more holistic strategy for managing CNLBP. By focusing on the key goals of this study—evaluating the impact of these interventions on core muscle strength, pain intensity, and functional capacity—the following discussion aims to provide a comprehensive understanding of their therapeutic effects and underlying mechanisms.

### 4.1 Muscle strength improvement

In muscle strength testing, this study has demonstrated that core stability training and combined breathing training have significantly enhanced core muscle strength in patients with CNLBP. The experimental group showed remarkable improvements in muscle strength in both forward flexion and backward extension directions, whereas the core stability training-only group achieved moderate gains, and the control group without intervention exhibited no statistically significant changes. These findings indicate that exercise interventions play a pivotal role in activating deep core muscles and enhancing core stability, with the addition of breathing training further amplifying these effects.

Core stability training has been shown to optimize the recruitment patterns of deep muscles such as the transversus abdominis and multifidus, enhancing neuromuscular control and improving dynamic lumbar stability (31). In the experimental group, the integration of breathing training likely contributed to further strengthening of core stability by increasing intra-abdominal pressure (IAP) and enhancing diaphragm function (32). This was reflected in the significant improvement in transversus abdominis strength, supporting the unique role of breathing training in promoting the coordinated activation of deep muscles (18).

The control group’s lack of significant improvement in muscle strength aligns with existing research, which suggests that core muscles remain underutilized in the absence of exercise stimuli, potentially due to pain inhibition mechanisms (1). Additionally, differences in strength improvements across the groups may also reflect variations in gender distribution within each group. It has been suggested that gender differences in muscle mass, hormonal profiles, and baseline core strength could influence responses to exercise interventions (33). Although, we did not specifically require balanced gender distribution across groups during participant allocation due to the relatively small sample size, this approach was necessary to ensure the feasibility of the intervention. Including gender as a stratification factor would have required stricter inclusion criteria, potentially leading to the exclusion of a substantial number of participants. Nevertheless, existing literature highlights that males may experience greater muscle hypertrophy due to higher testosterone levels, whereas females may exhibit more balanced neuromuscular control (21, 31).

These findings demonstrate the comprehensive benefits of combined interventions, particularly in multidirectional muscle strength improvement, and provide foundational evidence for their role in reducing pain scores and improving functionality in CNLBP patients. Future research should include subgroup analyses to investigate the effects of gender-specific training adaptations and examine how variations in baseline characteristics influence muscle strength outcomes.

### 4.2 Pain relief and VAS score reduction

The findings of this study revealed significant reductions in VAS scores for pain intensity among CNLBP patients who underwent core stability training and combined breathing training. The experimental group demonstrated a notably larger decrease in VAS scores compared to the core stability training-only group, while the control group showed minimal improvement. These results underline the effectiveness of exercise interventions in alleviating pain and suggest that the addition of breathing training provides unique benefits in pain management.

The reduction in VAS scores is closely linked to the improvement in core muscle strength observed in this study. Enhanced strength in deep core muscles, such as the transversus abdominis and multifidus, provides greater lumbar stability, reducing abnormal spinal movements and mitigating mechanical stress on the lumbar region—two key factors contributing to chronic pain (31, 34). Core stability training improves neuromuscular control, facilitating coordinated muscle recruitment and thereby decreasing the strain placed on pain-sensitive structures such as intervertebral disks and ligaments (33). These effects are further amplified by breathing training, which stabilizes intra-abdominal pressure (IAP), supports spinal alignment, and reduces compressive forces on the lumbar spine, leading to a decrease in pain perception (32).

Moreover, the observed discrepancy in VAS improvements between the experimental and core stability training-only groups highlights the additive value of breathing exercises. Breathing training not only enhances core muscle activation but also addresses secondary factors such as respiratory inefficiencies and postural imbalances, which are often overlooked in traditional core training programs (18). These synergistic effects likely contributed to the more pronounced pain relief in the experimental group.

While gender distribution was not balanced across groups due to the small sample size, existing literature suggests that gender-related differences in pain perception and muscle function might have influenced individual responses to the interventions (21, 33). For instance, hormonal and structural variations may affect the sensitivity of pain receptors and the ability to develop muscle strength, potentially explaining some of the variability in VAS outcomes. Future studies incorporating gender-specific analyses could provide deeper insights into these interactions.

The control group, which showed minimal improvements in both muscle strength and pain relief, underscores the necessity of active interventions for effective CNLBP management. Passive or non-intervention approaches fail to address the underlying muscular and neuromuscular dysfunctions, perpetuating the cycle of pain and disability (35).

Finally, the significant reductions in VAS scores observed in the experimental group pave the way for linking pain relief to functional improvements. Decreased pain intensity reduces the inhibitory effects of pain on daily activities, enabling patients to engage more actively in functional tasks. This lays the foundation for the subsequent discussion on how improved muscle strength and pain relief contribute to better functional outcomes, as reflected in ODI scores.

### 4.3 Functional improvement and ODI score reduction

The results of this study demonstrated significant improvements in functional outcomes, as measured by ODI scores, among CNLBP patients who underwent core stability training and combined breathing training. The experimental group exhibited the most pronounced reduction in ODI scores, followed by the core stability training-only group, while the control group showed negligible changes. These findings underscore the critical role of structured exercise interventions in improving functional capabilities and reducing disability in CNLBP patients.

The reduction in ODI scores is closely tied to the improvements in muscle strength and pain relief observed in this study. Enhanced core muscle strength provides greater spinal stability, which reduces biomechanical inefficiencies during daily activities and minimizes compensatory movements that often exacerbate functional limitations (36). Furthermore, the significant reduction in pain intensity, as reflected in VAS scores, likely enabled participants to perform activities of daily living with less discomfort, thereby contributing to better ODI outcomes. This aligns with previous research suggesting that pain relief can reduce fear-avoidance behaviors and encourage patients to re-engage in functional tasks (6, 22).

The experimental group’s superior performance compared to the single training group highlights the added value of breathing training in functional recovery. Breathing training enhances respiratory efficiency and intra-abdominal pressure (IAP), which are essential for stabilizing the spine during functional movements (20). Additionally, breathing exercises may indirectly improve proprioception and neuromuscular coordination, leading to better movement control and reduced functional impairment (37). These benefits were particularly evident in tasks requiring dynamic spinal stability, as reported in post-intervention functional assessments.

In contrast, the control group’s lack of significant improvement in ODI scores further emphasizes the importance of active interventions. Without targeted training, the underlying muscle imbalances and pain-related dysfunctions in CNLBP patients persist, perpetuating their functional limitations (34, 38).

Although gender distribution was not balanced across groups, potential gender-related differences in functional recovery should be considered. Existing literature suggests that females may demonstrate greater flexibility and postural control, which could positively influence ODI improvements, while males may benefit more from muscle hypertrophy and strength-oriented interventions (11, 20, 39, 40). These differences highlight the need for future studies to stratify participants by gender and evaluate its impact on functional outcomes, enabling more personalized rehabilitation strategies.

In conclusion, the combined intervention of core stability and breathing training proved to be the most effective in reducing functional disability, as indicated by ODI scores, outperforming both the single training approach and the control condition. By addressing both muscular and respiratory components, this comprehensive intervention not only alleviates pain but also facilitates meaningful improvements in daily functioning, making it a promising approach for CNLBP rehabilitation.

## 5 Limitations

Sample size and diversity: The small sample size may have limited the statistical power and generalizability of the findings. Additionally, the lack of consideration for demographic diversity, such as age and gender, could influence the applicability of the results to broader CNLBP populations.

Intervention duration and frequency: The 12-week intervention period may have been too short to capture long-term effects. Moreover, a standardized intervention frequency may not have accounted for individual variability, potentially impacting treatment outcomes.

Long-term effects and follow-up: The absence of long-term follow-up restricts the ability to assess the sustainability of improvements in strength, pain, and functionality. Evaluating long-term outcomes is essential to understanding the lasting impact of these interventions.

## 6 Conclusion

This study demonstrated that both 12-week core training and core training combined with breathing exercises significantly improved pain, lumbar function, and core strength in CNLBP patients. Among the two approaches, combined training consistently outperformed core training alone, highlighting the added value of incorporating breathing exercises to enhance intervention outcomes.

Future research should prioritize investigating the long-term effects of these integrated interventions to determine their sustainability and clinical impact. The use of advanced assessment tools, such as imaging and biomechanical analysis, could provide deeper insights into individual differences in response to treatment, allowing for more personalized exercise programs. Moreover, adopting an interdisciplinary approach that incorporates physical therapy, respiratory training, and psychological support could pave the way for novel and comprehensive treatment strategies for CNLBP, further improving patient outcomes and quality of life.

## Data Availability

The raw data supporting the conclusions of this article will be made available by the authors, without undue reservation.
